# Compliance with the enhanced recovery after surgery protocol and prognosis after colorectal cancer surgery: A prospective cohort study

**DOI:** 10.18632/oncotarget.18602

**Published:** 2017-06-22

**Authors:** Liang Li, Juying Jin, Su Min, Dan Liu, Ling Liu

**Affiliations:** ^1^ Department of Anesthesiology, The First Affiliated Hospital of Chongqing Medical University, Chongqing, China

**Keywords:** enhanced recovery after surgery, colorectal cancer, compliance, prognosis

## Abstract

We explored the effects of different levels of compliance with an enhanced recovery after surgery (ERAS) protocol on the short-term prognosis of patients who underwent colorectal cancer surgery. We conducted a single-center prospective cohort study in which 254 patients who received surgical treatment in a teaching tertiary care hospital were enrolled from March 2016 to November 2016. The patients were divided into four groups (I, II, III, and IV) based on individual compliance rates; the corresponding range of compliance rates was 0-60%, 60-70%, 70-80%, and 80-100%, and the number of patients in each group was 66, 63, 53, and 72, respectively. In the four groups from low to high compliance with ERAS (group I, II, III, and IV), the incidence of surgical site infections was 24.2%, 20.6%, 9.4%, and 6.9% (P < 0.05); the overall incidence of postoperative complications was 41.3%, 33.3%, 26.4%, and 16.7% (P < 0.05); the median length of postoperative hospital stay (in days) was 12.5, 10, 9, 8 (P < 0.05); and the median total hospital cost (Chinese Yuan) was 71,733, 73,632, 65,861, and 63,289 (P < 0.05), respectively. These results suggest that higher compliance with the ERAS protocol was associated with a lower incidence of surgical site infections, lower overall postoperative complication rate, shorter postoperative hospital stays, and lower total hospital costs.

## INTRODUCTION

Colorectal cancer is the second most common cancer in females and the third most common in males, with an estimated 0.69 million cancer-related deaths occurring in 2012 worldwide [1]. An increasing number of studies have confirmed that the enhanced recovery after surgery (ERAS) protocol has the advantages of reducing the length of postoperative hospital stay after colorectal surgery without compromising patient safety and even reduced the incidence of postoperative complications [2–6].

ERAS is a series of improvement measures used to guide optimization during the perioperative period and is based on the theory of evidence-based medicine. ERAS has been applied in many countries [7, 8]. Professors Kehlet and Wilemore formally promoted the concept of ERAS in 2001, and it is now widely used in the surgical field [9, 10]. Through the establishment of an ERAS team to promote multi-disciplinary cooperation, good clinical outcomes have been achieved in the forms of reduced incidence of postoperative complications and reduced postoperative hospital stays; these findings support the cost-effectiveness of ERAS [11–13]. The ERAS protocol of colorectal cancer surgery is the most widely used.

Several clinical studies have shown that the short-term and long-term prognoses of patients with colorectal cancer are closely related to the compliance rates with the ERAS protocol. However, the evidence is still not sufficient due to the limited number of items in the ERAS protocols in past studies [13–16].

In this prospective study, we analyzed the clinical data of patients with colorectal cancer who were treated according to the ERAS protocol. We also explored the effects of the ERAS protocol compliance rate on the short-term prognosis, such as postoperative complications and length of postoperative hospital stay.

## RESULTS

### Participants

**Figure 1:**
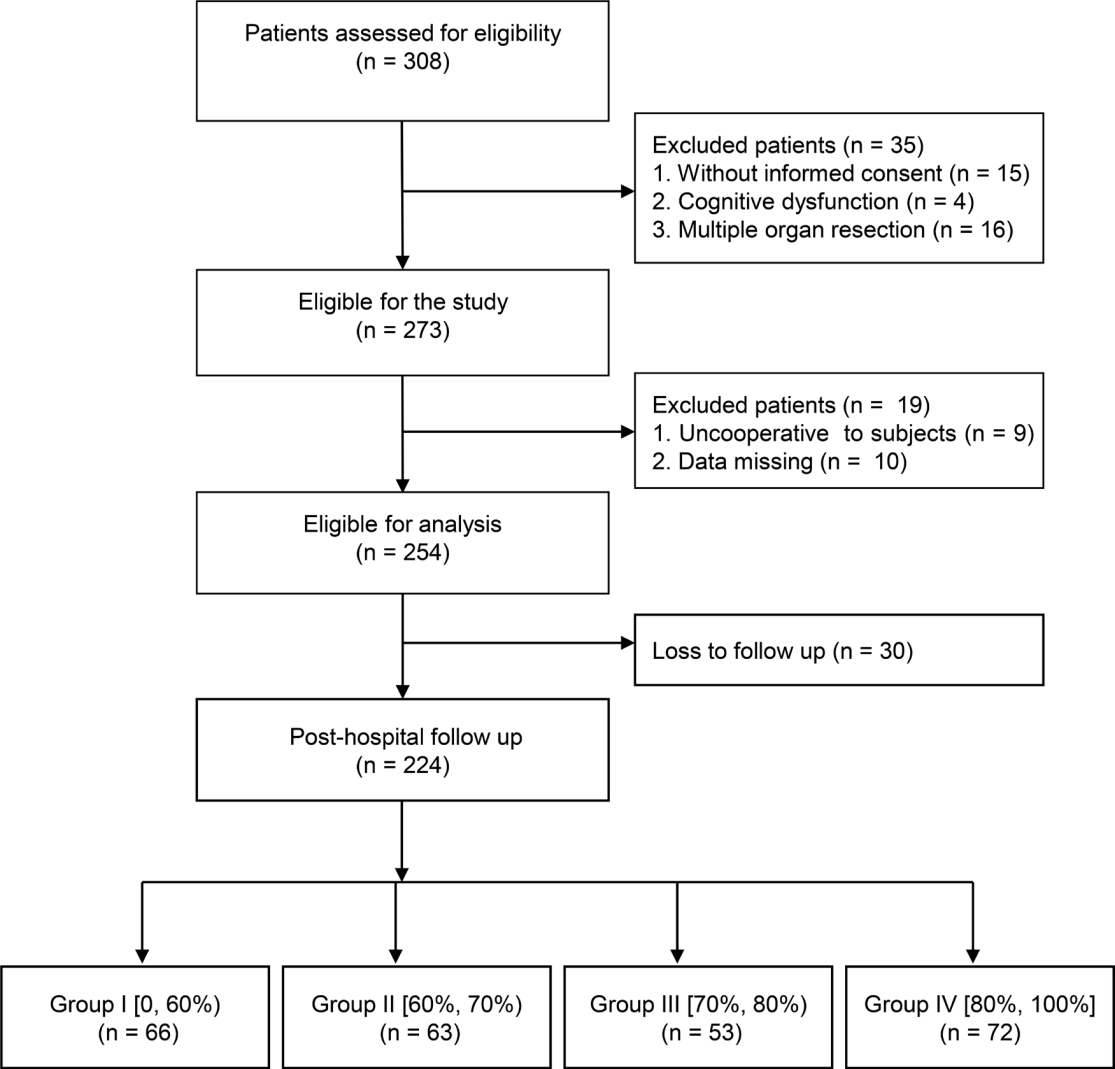
Flowchart of the study participant selection

A total of 273 patients participated in the study. Nineteen patients dropped out during the study period, leaving data from 254 patients for short-term outcome analyses. Furthermore, 30 patients were lost during the post-discharge follow-up, allowing for long-term outcomes analyses of 224 patients (Figure 1). Patients were divided into four groups according to their compliance with the ERAS protocol (Table 1). Group I included patients with compliance less than 60%; group II, those with 60% to 70% compliance; group III, patients with 70% to 80% compliance; and group IV, patients with more than 80% compliance. The number of patients in each group was 66, 63, 53, and 72, respectively. Demographic characteristics and perioperative data are shown in Table 2. The compliance rate of ERAS-related items among the four groups is presented in Table 3.

**Table 1:** ERAS protocol applied in the study

1. Preoperative counseling, patient education
2. Nutritional assessment and enteral nutrition (Supportan or Fresubin 500 ml) support
3. Cardiopulmonary function evaluation and optimization
4. No preoperative bowel preparation
5. Preoperative fasting time: 6-8 hours for solid food, 2 hours for clear liquids
6. Oral intake of 400 ml carbohydrate drink: up to 2-3 hours before the induction of anesthesia (10% glucose solution)
7. Intravenous antibiotics (cefoxitin 1.5 g or ceftriaxone 1 g) 30 minutes before incision
8. No preanesthetic medication
9. General anesthesia with rapid short-acting agents combined with TAP block
10. Laparoscopic surgery
11. Anesthesia depth monitoring with bispectral index or narcotrend index
12. Intraoperative lung-protective ventilatory strategy
13. Intraoperative neuromuscular monitoring
14. Prevention of intraoperative hypothermia
15. Intraoperative goal-directed fluid therapy and postoperative restrictive fluid administration
16. Perioperative blood glucose control
17. Multimodal prevention of PONV (5-HT3 receptor antagonist + dexamethasone + haloperidol)
18. Multimodal prevention of DVT (physical prophylaxis combined with low molecular weight heparin administration)
19. No nasogastric tube postoperatively
20. Prevention of stress ulcer (perioperative administration of proton pump inhibitor)
21. Multimodal management of postoperative pain (PCIA, TAP, NSAIDs, COX-2 inhibitor)
22. Avoiding incision infection
23. Early oral intake (drink water 2 hours after surgery, oral nutritional supplements on the first day after surgery, semi-solid diet on the second day after surgery)
24. Early mobilization (out-of-bed activity for 2 hours on the first postoperative day and 4-6 hours from the second postoperative day to discharge)
25. Removal of drainage tubes within three days after surgery
26. Removal of urinary catheter as soon as possible (within 24 hours for colon surgery patients; within 48 hours for rectal surgery patients)

TAP: transverse abdominis plane; PONV: postoperative nausea and vomiting; DVT: deep vein thrombosis; PCIA: patient-controlled intravenous analgesia; NSAIDs: nonsteroidal anti-inflammatory drugs.

**Table 2:** Demographic characteristics and perioperative data

	I [0, 60%)	II [60%, 70%)	III [70%, 80%)	IV [80%, 100%]	P value
**Patients (n)**	66	63	53	72	
**Age (year), median (IQR)**	65 (53, 75)	66 (54, 77)	64 (50, 71)	63.5 (52, 74)	0.300
**Sex (male/female)**	39/27	31/32	31/22	39/33	0.644
**BMI (kg/m2), (mean ± SD)**	22.8 ± 2.9	21.4 ± 3.2	23.1 ± 3.0	22.4 ± 3.1	0.014
**ASA grade**					0.042
II	39	39	41	56	
III	26	24	12	15	
IV	1	0	0	1	
**Diabetes mellitus**	6	9	8	5	0.384
**Hypertension**	21	19	12	22	0.702
**CHD**	8	7	4	3	0.313^*^
**COPD**	3	7	5	5	0.655^*^
**General anaesthesia/combined TAP block**	51/15	50/13	22/31	12/60	< 0.001
**Site of procedure (colon/rectal)**	41/25	43/20	36/17	58/14	0.113
**Preoperative hemoglobin (g/L), (mean ± SD)**	121.4 ± 23.2	117.3 ± 21.6	122.2 ± 22.9	116.0 ± 23.8	0.356
**Length of operation (min), median (IQR)**	230 (200, 293)	235 (215, 275)	255 (187, 315)	240 (192, 279)	0.706
**Intraoperative blood loss (ml), median (IQR)**	100 (50, 200)	100 (50, 200)	100 (50, 100)	80 (30, 100)	0.003
**Intraoperative net fluid input (ml), (mean ± SD)**	1897.7 ± 680.7	1732.2 ± 658.9	1555.7 ± 637.9	1538.3 ± 575.9	0.004

BMI: body mass index; ASA: American Society of Anesthesiologists; CHD: coronary heart disease; COPD: chronic obstructive pulmonary disease; IQR: interquartile range; SD: standard deviation.

^*^Fisher exact test, all other statistics: Chi-Square test.

**Table 3:** Comparison of compliance in the individual items of ERAS protocol

	Total	I [0, 60%)	II [60%, 70%)	III [70%, 80%)	IV [80%, 100%]	P value
**Patients (n)**	254	66	63	53	72	
**Education and counselling**	254 (100%)	66 (100%)	63 (100%)	53 (100%)	72 (100%)	
**Nutritional assessment and support**	119 (46.9%)	20 (30.3%)	26 (41.3%)	26 (49.1%)	47 (65.3%)	< 0.001
**Cardiopulmonary function evaluation**	214 (84.3%)	39 (59.1%)	54 (85.7%)	49 (92.5%)	72 (100%)	< 0.001
**No bowel preparation**	35 (13.8%)	4 (6.1%)	4 (6.3%)	7 (13.2%)	20 (27.8%)	< 0.001
**Forbidden to drink and eat**	207 (81.5%)	40 (60.6%)	52 (82.5%)	46 (86.8%)	69 (95.8%)	< 0.001
**Carbohydrate drinks**	154 (60.5%)	15 (22.7%)	29 (46.0%)	42 (79.2%)	68 (94.4%)	< 0.001
**Antibiotics prophylaxis**	254 (100%)	66 (100%)	63 (100%)	53 (100%)	72 (100%)	
**No premedication**	231 (90.9%)	53 (80.3%)	59 (93.7%)	51 (96.2%)	68 (94.4%)	0.013^*^
**Anesthesia protocols**	150 (59.1%)	23 (34.8%)	35 (55.6%)	32 (60.4%)	60 (83.3%)	< 0.001
**Laparoscopic surgery**	198 (78.0%)	43 (65.2%)	48 (76.2%)	42 (79.2%)	65 (90.3%)	0.005
**Depth of anesthesia**	229 (90.2%)	52 (100%)	58 (100%)	51 (100%)	68 (100%)	0.004
**Ventilation management**	225 (88.6%)	46 (69.7%)	59 (93.7%)	50 (94.3%)	70 (97.2%)	< 0.001
**Muscle relaxant**	109 (42.9%)	14 (21.2%)	22 (34.9%)	28 (52.8%)	45 (62.5%)	< 0.001
**Active warming**	200 (78.7%)	43 (65.2%)	44 (69.8%)	45 (84.9%)	68 (94.4%)	< 0.001
**Perioperative fluid management**	126 (49.6%)	15 (22.7%)	28 (44.4%)	33 (62.3%)	50 (69.4%)	< 0.001
**Control blood glucose**	210 (82.7%)	41 (62.1%)	53 (84.1%)	47 (88.7%)	69 (95.8%)	< 0.001
**PONV prophylaxis**	223 (87.8%)	46 (69.7%)	57 (90.5%)	49 (92.5%)	71 (98.6%)	< 0.001
**Thrombo-prophylaxis**	218 (85.8%)	45 (68.2%)	55 (87.3%)	49 (92.5%)	69 (95.8%)	< 0.001
**No nasogastric tube**	237 (93.3%)	60 (90.9%)	58 (92.1%)	48 (90.6%)	71 (98.6%)	0.127^*^
**Prevention of stress ulcer**	233 (91.7%)	58 (87.9%)	57 (90.5%)	48 (90.6%)	70 (97.2%)	0.170^*^
**Multimodal analgesic approaches**	132 (52.0%)	15 (22.7%)	25 (39.7%)	33 (62.3%)	59 (81.9%)	< 0.001
**Incision management**	226 (89.0%)	49 (74.2%)	56 (88.9%)	50 (94.3%)	71 (98.6%)	< 0.001
**Early oral intake**	113 (44.5%)	16 (24.2%)	17 (27.0%)	25 (47.2%)	55 (76.4%)	< 0.001
**Early mobilisation**	119 (46.9%)	12 (18.2%)	19 (30.2%)	28 (52.8%)	60 (83.3%)	< 0.001
**Remove drainage tubes**	92 (36.2%)	6 (9.1%)	10 (15.9%)	23 (43.4%)	53 (73.6%)	< 0.001
**Remove urinary catheter**	41 (16.1%)	1 (1.5%)	3 (4.8%)	12 (22.6%)	25 (34.7%)	< 0.001

^*^Fisher exact test, all other statistics: Chi-Square test.

### Postoperative complications

**Figure 2:**
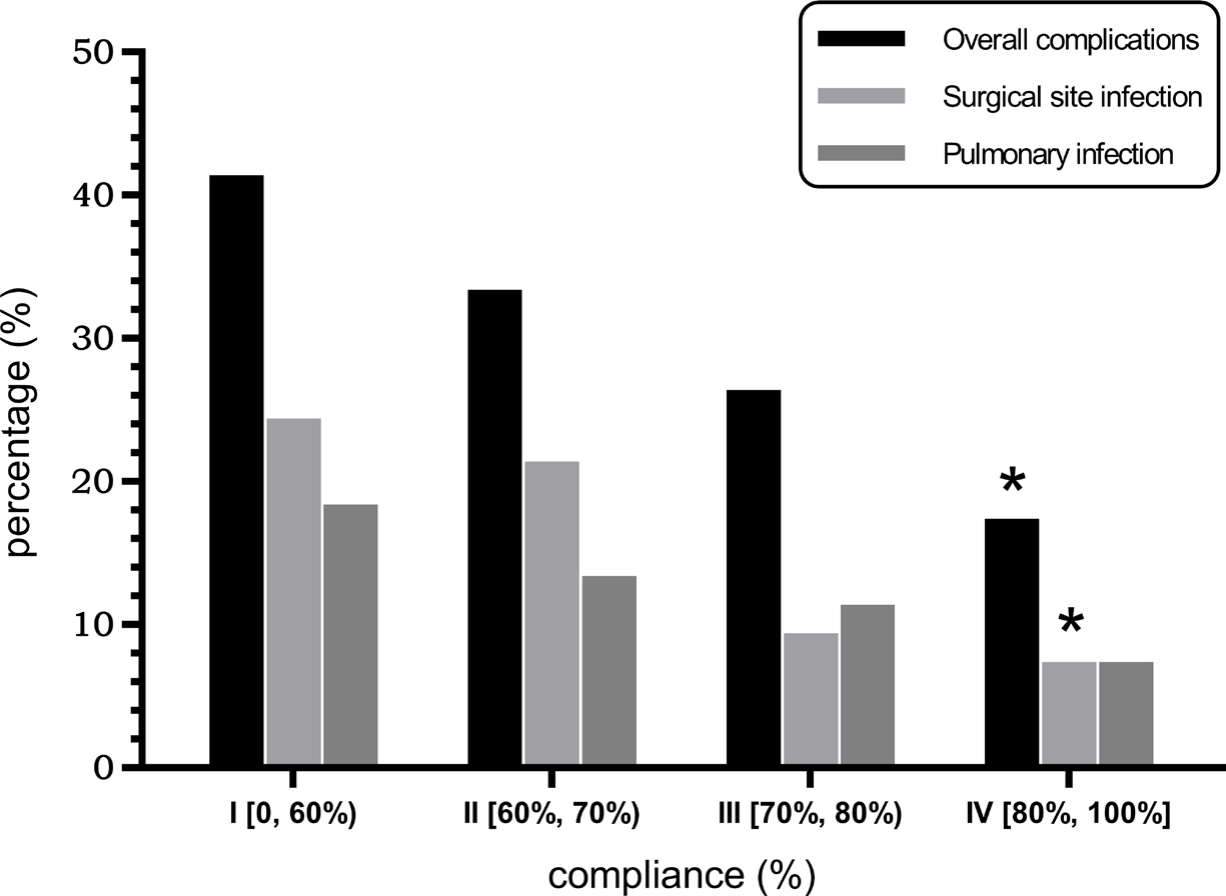
Association between the compliance to the ERAS protocol and the incidence of postoperative complications. “^*^” indicates a significant difference compared to group I (P < 0.05), and the P value was corrected using Bonferroni’s method

The incidence of postoperative complications for each group is shown in Table 4. The overall incidence of postoperative complications was 41.3%, 33.3%, 26.4%, and 16.7% in the four groups respectively (P = 0.023), with a significant difference between groups I and IV. Based on compliance rates, the incidence of surgical site infections (SSIs) was 24.2%, 20.6%, 9.4%, and 6.9% in the four groups of patients from low to high compliance (I, II, III, and IV), respectively (P = 0.013), with a significant difference between groups I and IV. The incidence of postoperative pulmonary infections was 18.2%, 12.7%, 11.3%, and 6.9% (P = 0.250) (Figure 2). There were no significant differences in the incidences of other specific postoperative complications between the four groups (P > 0.05). No patients experienced unplanned reoperations or died in the hospital.

**Table 4:** The incidence of postoperative complications

	Total	I [0, 60%)	II [60%, 70%)	III [70%, 80%)	IV [80%, 100%]	P value
**Patients (n)**	254	66	63	53	72	
**Acute heart failure**	1	0	0	1	0	0.209^*^
**Acute pancreatitis**	1	1	0	0	0	0.717^*^
**Acute renal failure**	0	0	0	0	0	
**Acute respiratory failure**	1	0	0	1	0	0.209^*^
**Anastomotic fistula**	2	2	0	0	0	0.170^*^
**Atrial fibrillation**	2	0	2	0	0	0.104^*^
**Cardiac arrest**	0	0	0	0	0	
**DVT**	5	2	1	1	1	0.933^*^
**Gastrointestinal bleeding**	2	2	0	0	0	0.170^*^
**Hoarseness**	1	0	0	1	0	0.224^*^
**Ileus**	6	2	1	2	1	0.933^*^
**Liver dysfunction**	5	1	3	1	0	0.233^*^
**Myocardial infarction**	1	1	0	0	0	0.717^*^
**Non-planned re-operation**	1	0	0	0	1	> 0.999^*^
**Persistent coma postoperatively ≥ 24 hours**	0	0	0	0	0	
**Pneumothorax**	0	0	0	0	0	
**POCD**	0	0	0	0	0	
**Postoperative reintubation**	0	0	0	0	0	
**Pulmonary infarction**	1	1	0	0	0	0.717^*^
**Pulmonary infections**	33	12	8	6	5	0.250
**Septicopyemia**	3	1	0	1	1	0.890^*^
**Septic shock**	0	0	0	0	0	
**SSIs**	39	16	13	5	5	0.013
**Stroke**	0	0	0	0	0	
**Urinary tract infections**	4	1	3	0	0	0.086^*^
**Wound dehiscence**	1	0	1	0	0	0.457^*^
**Overall complications**	73	26	21	14	12	0.023

POCD: postoperative cognitive dysfunction; SSIs: surgical site infections.

^*^Fisher exact test, all other statistics: Chi-Square test.

### Length of postoperative hospital stay and hospital costs

**Figure 3:**
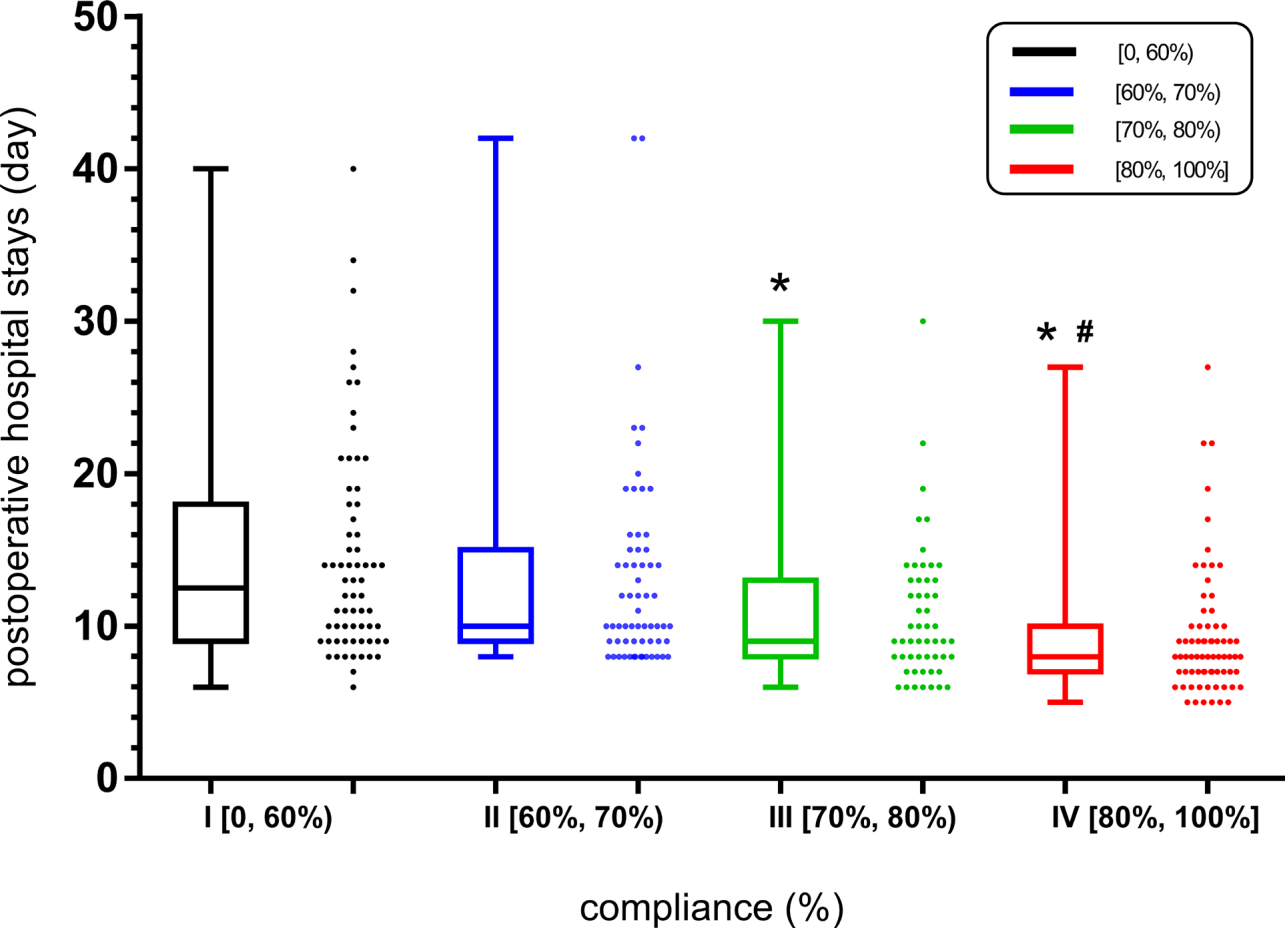
Association between the compliance rate of the ERAS protocol and the length of postoperative hospital stay. “^*^” indicates a significant difference compared to group I; “#” indicates a significant difference compared to group II. Analyzed using the Kruskal-Wallis test, P < 0.05

From low to high ERAS compliance rates, the length of postoperative hospital stay in days was 12.5 (interquartile range (IQR), 9-18), 10 (IQR, 9 - 15), 9 (IQR, 8 - 13), and 8 (IQR, 7 - 10) in groups I, II, III and IV, respectively (P < 0.001), as shown in Figure 3. There were significant differences between groups I and III, between groups I and IV, and between groups II and IV, respectively (P < 0.05).

The total hospital costs (Chinese Yuan) were 71,733 (IQR, 62136 - 88224), 73,632 (IQR, 63277 - 81804), 65,861 (IQR, 582723 - 74607), 63,289 (IQR, 55721 - 70418) for groups I to IV, (P < 0.001). There were significant differences between groups I and IV, and between groups II and IV (P < 0.05).

In addition, there were no statistically significant differences in postoperative 30-day readmission rate, unplanned reoperation rate, in-hospital mortality rate, incidence of chronic pain, or mortality within 90 postoperative days between the four groups (P > 0.05).

### Post-discharge follow-up

Patients were contacted by telephone at 30 and 90 days following surgery. There were no significant differences regarding postoperative 30-day readmission rate, incidence of chronic pain, or mortality within 90 days after surgery between the four groups (P < 0.05). A total of 14 patients (5.5%) were readmitted within 30 days (3, 5, 3, and 3 patients in groups I, II, III, and IV, respectively). All readmitted patients received an effective surgical operation or conservative treatment (Table 5).

**Table 5:** Post-discharge follow-up

	Total	I [0, 60%)	II [60%, 70%)	III [70%, 80%)	IV [80%, 100%]	P value
**Patients (n)**	254	66	63	53	72	
**Follow-up**	224	58	53	47	66	0.604
**Readmission (30 days)**	14	3	5	3	3	0.722^*^
**Chronic pain (90 days)**	41	10	10	8	13	0.979
**Dead (90 days)**	3	1	0	2	0	0.128^*^

^*^Fisher exact test, all other statistics: Chi-Square test.

## DISCUSSION

The rate of compliance with the ERAS protocol was associated with the short-term prognosis of patients. To improve clinical outcomes, monitoring compliance with ERAS protocol was essential [17]. In agreement with other studies, a high compliance with the ERAS protocol was associated with reduction in both the overall incidence of postoperative complications and the length of postoperative hospital stay. The difference in overall postoperative complication rates between the highest compliance groups was 24.6%. Several studies have also shown that the improved compliance with the ERAS protocol was beneficial to postoperative outcomes [14, 18–20]. Moreover, we observed no significant differences in 30-day readmissions among the four groups. This finding is consistent with the results of a recent meta-analysis of ERAS applications following colorectal cancer surgery [6].

There were significant differences in some demographic characteristics and perioperative variables between the four groups, such as type of anesthesia, intraoperative blood loss, intraoperative net fluid input, and American Association of Anesthesiologists (ASA) grade; these differences may have influenced the incidence of postoperative complications. Higher ASA grades lead to higher rates of postoperative complications [21]. Differences in type of anesthesia type, intraoperative blood loss and intraoperative net fluid input could be explained by different anesthesia protocols, surgical approaches, and individualized perioperative fluid management in the ERAS protocol.

For some reasons, the compliance rates differed greatly between different patient groups. The concept of ERAS has been introduced and implemented at our institution, and the members of the multidisciplinary team (MDT) had got full coordination and cooperated more closely over time. However, to ensure patient safety, we also respected the patients’ independent choices. In the lowest compliance group, items with lower implementation rates included carbohydrate drinks, anesthesia protocols, perioperative fluid management, multimodal analgesic approaches, early oral intake, early mobilization, early removal of drainage tubes, and early removal of urinary catheter. These interventions are seldom or not all implemented during traditional perioperative management due to ingrained theory, which may lead to a poor prognosis in patients undergoing colorectal cancer surgery [22–28].

It may be difficult to compare our current research with other studies because of the differences in the number of ERAS items and the definition of some items. In our opinion, an increase in the number of ERAS protocol items is very important to improving patient outcomes, which are associated with the compliance rate of each individual.

The ERAS protocol in our study contained a total of 26 items, which is a relatively large number of implementation items. Some items were not included in most previous studies, such as nutritional assessment and support, cardiopulmonary functional evaluation and optimization, preoperative fasting instruction, no preanesthetic medications, anesthesia protocols, and anesthesia depth monitoring, and so on. We believe that 26 items would be more sufficient for assessing the association between compliance and prognosis. Severe malnutrition is known to increase the incidence of postoperative complications after major abdominal surgery [29]. As a basic clinical method to evaluate respiratory, the application of cardiovascular and metabolic functions, cardiopulmonary exercise tests have become increasingly widespread [30]. Additionally, fasting overnight can cause postoperative insulin resistance and discomfort of the patients. According to the latest ASA guidelines, for adults having an elective surgical procedure, limited non-fatty solid food may be consumed up to 6 hours prior to anesthesia, and drinking clear fluids is encouraged up to 2 hours before anesthesia [31–32]. The preoperative use of antianxiety drugs in traditional clinical routine may decrease the difficulty of postoperative pain management. However, preoperative administration of antianxiety drugs could lead to delayed recovery from anesthesia, which is unfavourable to the prognosis [33]. Emerging evidence indicates deep anesthesia is harmful and may increase the risk of postoperative delirium. Use of bispectral index monitoring may help determine the depth of anesthesia to apply in patients [34]. Ultrasound-guided transversus abdominis plane block can significantly reduce the consumption of opioid drugs and opioid-associated side effects in a short time postoperatively [35]. We think it is necessary to include these items in the ERAS protocol. Meanwhile, the detailed content of some ERAS items was inconsistent among different studies, such as the time to removal of drainage tubes and urinary catheter, and perioperative fluid management [14, 15, 20].

We did not adopt the method of grouping according to the time phase of ERAS protocol implementation [13–15]. To control for factors that may change over time, patients were grouped directly according to the range of compliance rate to improve the accuracy of the conclusion. We also focused on the incidence of SSIs and postoperative pulmonary infections, the total hospital costs, and the incidence of chronic pain within 90 days after surgery. The incidence of SSIs, which was reduced by 17.3%, was effectively controlled. The total hospital costs were also obviously decreased. The rate of postoperative pulmonary infections showed a decreasing trend, with an increase in compliance rate. With larger sample sizes, the difference in postoperative pulmonary infection rates between the four groups may be significant. In addition, there were no significant difference in the incidence of chronic pain within 90 days after surgery between the four groups.

A few limitations of our study should be mentioned. First, the implementation rate of some items in the ERAS protocol, such as no preoperative bowel preparation and early removal of urinary catheter, was generally not high enough. Strong evidence indicates that close multidisciplinary cooperation is essential to improve the implementation of ERAS protocols. Second, the significant difference in ASA grade (P = 0.042) between the four groups may affect the reliability of the outcomes. Third, this was a single-center observational study, multicenter and large-scale trials are clearly needed to verify the current results. In the future, the association between increased compliance with the ERAS protocol and improved long-term outcomes following colorectal surgery warrants further investigation. Moreover, a specific ERAS protocol for either colon cancer or rectal cancer surgery should be developed to provide patients with individualized perioperative care.

In conclusion, higher compliance with the ERAS protocol was associated with lower incidence of SSIs, lower overall postoperative complication rate, shorter postoperative hospital stay, and lower total hospital costs.

## MATERIALS AND METHODS

### Study design and patient selection

This prospective observational cohort study was registered with ClinicalTrials.gov (NCT02728973) and approved by the local Ethics Committee. Patients who were scheduled for elective colon or rectal resection at our institution from March 2016 to November 2016 were recruited. Patients were informed of the ERAS protocol and signed the written informed consent form on the day of admission. Inclusion criteria were age greater 18 years old and elective open or laparoscopic colorectal surgery. Exclusion criteria were cognitive dysfunction, multiple organ resection, uncooperative subjects, or failure to obtain informed consent. All surgeries and anesthesia were performed by the same group of surgeons and anesthesiologists.

Five surgeons, four anesthesiologists, eleven nurses, two physiotherapists, and two dieticians formed the ERAS MDT, which effectively implemented the ERAS protocol. Everyone on the team reported their work and communicated with each other at weekly meetings to ensure that the protocol was running well. The ERAS protocol included a total of 26 items which was composed of preoperative, intraoperative and postoperative interventions, as presented in Table 1. We recorded the implementation of each item for each patient. Compliance rate was calculated as the number of perioperative interventions fulfilled from the 26 items ERAS protocol. Patients were divided into four groups according to their compliance rate with the ERAS protocol.

Demographic characteristics included age, gender, ASA grade, type of anesthesia, body mass index (BMI), preoperative hemoglobin and site of procedure. Comorbidities such as diabetes mellitus, hypertension, coronary heart disease (CHD) and chronic obstructive pulmonary disease (COPD) were also recorded. Intraoperative data including length of operation, intraoperative blood loss and net fluid input were collected. Patients were followed by the ERAS team members during hospitalization. All of the relevant clinical data were recorded over time.

All patients were discharged if they met the following discharge criteria: no intravenous fluids, no signs of infection, ability to tolerate solid food, passage of first flatus or first stool, adequate pain control with oral analgesics, and ability to ambulate independently. Patients were contacted by telephone at 30 and 90 days following surgery. Data on readmission and occurrence of chronic postoperative complication were collected.

### Outcomes

The primary outcome measure was the overall incidence of postoperative complications within 30 postoperative days. Secondary outcome measures were the specific incidences of postoperative complications, such as SSIs, ileus, atrial fibrillation, pulmonary infection, postoperative length of hospital stay, total hospital costs, in-hospital mortality, readmission rate within 30 days post-discharge, incidence of chronic postoperative pain and postoperative mortality within 90 postoperative days.

### Statistical analyses

All variable data were descriptively analyzed via SPSS for Windows version 17.0. All data were presented as the means ± standard deviation (SD), medians (25th percentile to 75th percentile), or counts (percentages), as appropriate. Continuous data were compared using analysis of variance or the Kruskal-Wallis test in terms of data distribution. For categorical variables, comparison of groups was performed with chi-square test or Fisher’s exact test. Data were considered statistically significant at P < 0.05.

## Abbreviations

ERAS: Enhanced recovery after surgery
MDT: multi-disciplinary team
SSIs: surgical site infections
IQR: interquartile range
BMI: Body mass index
ASA: American society of anesthesiologists
COPD: chronic obstructive pulmonary disease
CHD: coronary heart disease
SD: Standard deviation
SPSS: Statistical package for the social sciences
TAP: transverse abdominis plane
PCIA: patient-controlled intravenous analgesia
DVT: deep vein thrombosis
PONV: postoperative nausea and vomiting
NSAIDs: nonsteroidal anti-inflammatory drugs
