# Correction: Compliance with the enhanced recovery after surgery protocol and prognosis after colorectal cancer surgery: A prospective cohort study

**DOI:** 10.18632/oncotarget.22102

**Published:** 2017-10-27

**Authors:** Liang Li, Juying Jin, Su Min, Dan Liu, Ling Liu

**Affiliations:** ^1^ Department of Anesthesiology, The First Affiliated Hospital of Chongqing Medical University, Chongqing, China

**This article has been corrected:** the funding information statement was updated and is as follows:

**FUNDING**

This work was supported by grant of the Municipal Medical Key Discipline of Chongqing (YuWeiKeJiao No. 2007-2), and by grant of Integrated Demonstration Project of Chongqing Science and Technology Commission (cstc2015jcsf10008-1).

Original article: Oncotarget. 2017; 8:53531-53541. https://doi.org/10.18632/oncotarget.18602

